# RhoGDI deficiency induces constitutive activation of Rho GTPases and COX-2 pathways in association with breast cancer progression

**DOI:** 10.18632/oncotarget.5416

**Published:** 2015-09-25

**Authors:** William P. Bozza, Yaqin Zhang, Kory Hallett, Leslie A. Rivera Rosado, Baolin Zhang

**Affiliations:** ^1^ Office of Biotechnology Products, Center for Drug Evaluation and Research, Food and Drug Administration, Silver Spring, MD 20993, USA; ^2^ United States Public Health Service Commissioned Corps, Rockville, MD 20852, USA

**Keywords:** RhoGDI, breast cancer, tumor growth, Rho GTPases, COX-2 activation

## Abstract

Rho GDP Dissociation Inhibitor (RhoGDI) is a key regulator of Rho GTPases. Here we report that loss of RhoGDI significantly accelerated xenograft tumor growth of MDA-MB-231 cells in animal models. At the molecular level, RhoGDI depletion resulted in constitutive activation of Rho GTPases, including RhoA, Cdc42, and Rac1. This was accompanied by Rho GTPase translocation from the cytosol to membrane compartments. Notably, COX-2 protein levels, mRNA expression, and biological activity were markedly increased in RhoGDI-deficient cells. The upregulated expression of COX-2 was directly associated with increased Rho GTPase activity. Further, we assessed the expression level of RhoGDI protein in breast tumor specimens (*n* = 165) by immunohistochemistry. We found that RhoGDI expression is higher in the early stages of breast cancer followed by a significant decrease in malignant tumors and metastatic lesions (*p* 0.01). These data suggest that downregulation of RhoGDI could be a critical mechanism of breast tumor development, which may involve the hyperactivation of Rho GTPases and upregulation of COX-2 activity. Additional studies are warranted to evaluate the therapeutic potential of inhibiting Rho GTPases and COX-2 for treating breast cancers.

## INTRODUCTION

The Rho family of small GTPases (e.g., Rac1, Cdc42, and RhoA) are molecular switches that transduce extracellular signals to downstream effectors. These proteins control multiple signaling pathways that are essential for normal cellular functions. Specifically, Rho GTPases promote actin organization, cell motility, polarity, growth, survival, and gene transcription [1]. However, Rho GTPase expression and activity is often deregulated in many human tumors, contributing to several aspects of malignant phenotypes including uncontrolled cell growth, angiogenesis, and invasive phenotypes [2].

As molecular switches, Rho GTPases cycle between GDP-bound (inactive, off) and GTP-bound (active, on) states. The GDP/GTP cycle is tightly regulated by multiple protein families, including guanine nucleotide exchange factors (RhoGEFs), GTPase-activating proteins (RhoGAPs), and Rho GDP dissociation inhibitors (RhoGDIs). While GEFs stimulate GDP exchange for GTP, GAPs catalyze GTP hydrolysis to GDP. RhoGDIs add an additional layer of regulation by controlling Rho GTPase subcellular localization and their physical interactions with GEF or GAP proteins, thereby dictating spatial and temporal activation patterns of Rho GTPases [3]. In doing this RhoGDIs form stable complexes with individual Rho proteins. To date there are three human RhoGDIs that have been identified, including RhoGDI (RhoGDI-1or RhoGDI-α), D4-GDI (RhoGDI-2 or RhoGDI-β), and RhoGDI-3. Together they are responsible for the regulation of the entire Rho GTPase family consisting of at least 22 members. RhoGDI is the founding member and binds to all Rho GTPases that have been examined (e.g., Rac1, Cdc42, and RhoA). By contrast, D4-GDI preferentially binds to Rac1 subfamily members [4]. Increasing evidence shows that RhoGDI protein expressions are aberrantly regulated in cancer cells when compared to normal counterparts. The altered expression of RhoGDI protein is expected to directly impact Rho GTPases activity and downstream signaling cascades. However, conflicting results have been reported for RhoGDI expression in breast cancer cells [5, 6]. As a result the exact role of RhoGDI as a promoter or suppressor of breast cancer progression remains elusive.

In this work we approached the issue through knockdown of RhoGDI in MDA-MB-231 human breast cancer cells. In xenograft mouse models, RhoGDI-deficient cells grew into tumors at a dramatically increased rate compared to parental cells or cells expressing a control siRNA. Consistently, RhoGDI protein expression in primary breast tumors (*n* = 165) was found to be significantly decreased during tumor progression from benign to malignant and metastatic lesions. At the molecular level, RhoGDI knockdown resulted in constitutive activation of multiple Rho GTPases (e.g., RhoA, Rac1, and Cdc42) and also led to a concomitant upregulation of COX-2 expression and activity. As upregulated COX-2 activity is widely implicated in cancer cell growth and invasion [7–11], our data provides a possible link between downregulation of RhoGDI and subsequent activation of COX-2 in promoting breast cancer. This work also suggests that Rho GTPase and COX-2 inhibition could be explored as a therapy for treating advanced breast tumors.

## RESULTS

### Targeted knockdown of RhoGDI in MDA-MB-231 breast cancer cells increases xenograft tumor growth in mouse models

**Figure 1:**
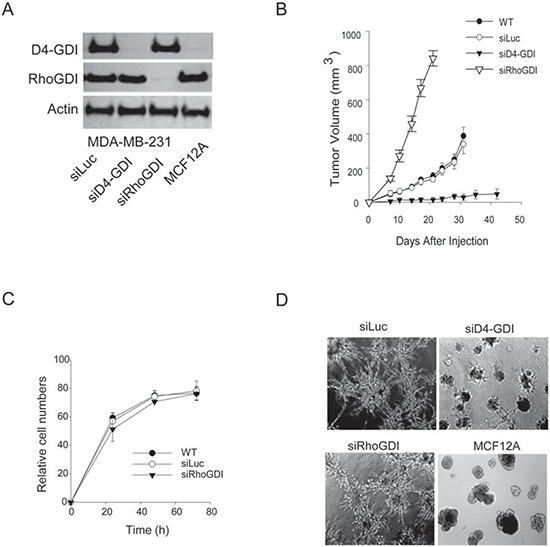
Effects of RhoGDI knockdown *in vivo* and *in vitro* **A.** Immunoblots of RhoGDI and D4-GDI protein expression in stable MB-231 cell lines transfected with a pRNA-6.1 plasmid expressing siRNA specific to firefly luciferase (siLuc), RhoGDI (siRhoGDI), or D4-GDI (siD4-GDI) transcript (see Methods). MCF12A, a normal breast epithelial cell line, was also analyzed as a control. **B.** Athymic nude mice were subcutaneously injected with indicated cell lines (5 × 10^6^ cells) and monitored for tumor growth. Each curve shows tumor volumes in cubic millimeters (mean ± S.D.; *n* = 10). *p < 0.01 compared with parental cells (WT). **C.** Comparison of cell proliferation rates *in vitro*. Cells (3 × 10^3^) were plated in 96-well plates and live cell numbers were determined by MTS assay at various time points. **D.** Phase-contrast microscopy of cells (2 × 10^4^) cultured onto 1 mm thick Matrigel for 12 days in eight chamber slides. Solidified Matrigel was covered with complete growth medium and incubated at 37°C and 10% CO_2_ in air. Representative images from three independent experiments are shown.

To assess the role of RhoGDI in breast cancer progression, we generated a stable MDA-MB-231 breast cancer cell line in which RhoGDI expression is depleted. This was achieved by transfection of a pRNA-U6.1 plasmid which synthesizes small interfering RNA (siRNA) specific to human RhoGDI transcript (siRhoGDI) or to firefly luciferase (siLuc) as a negative control [12]. The MB-231 cell line was chosen because it has been widely used as a model system for studying the molecular basis of human breast cancer cell growth and invasion [13]. Stable clones expressing siRhoGDI were confirmed to be deficient in the expression of RhoGDI but retained expression of the homologous D4-GDI family member (Fig. 1A). Strikingly, analysis of tumor xenograft growth of the stable cell lines after subcutaneous injection into athymic nude mice revealed that RhoGDI-depleted cells grew into a tumor at a significantly higher rate than siLuc and parental control cells (Fig. 1B). This effect is in sharp contrast to knockdown of D4-GDI, which was shown to suppress tumor growth of MDA-MB-231 cells [4]. RhoGDI and D4-GDI appear to play opposing roles in breast cancer progression. Surprisingly, RhoGDI depletion had no effect on cell proliferation when cells were grown as a monolayer on plastic dishes (Fig. 1C). Also the invasive phenotype of MB-231 cells when grown on Matrigel was retained for siRhoGDI cells (Fig. 1D). The accelerated tumor growth of siRhoGDI xenografts likely involves biological factors that are not present under the *in vitro* culture conditions. In this regard, it is well documented that the tumor microenvironment can bestow important traits and characteristics to cancer cells which can often be absent in conventional monolayer cell cultures[14].

### Rho GTPases are constitutively activated in RhoGDI-deficient cells

**Figure 2:**
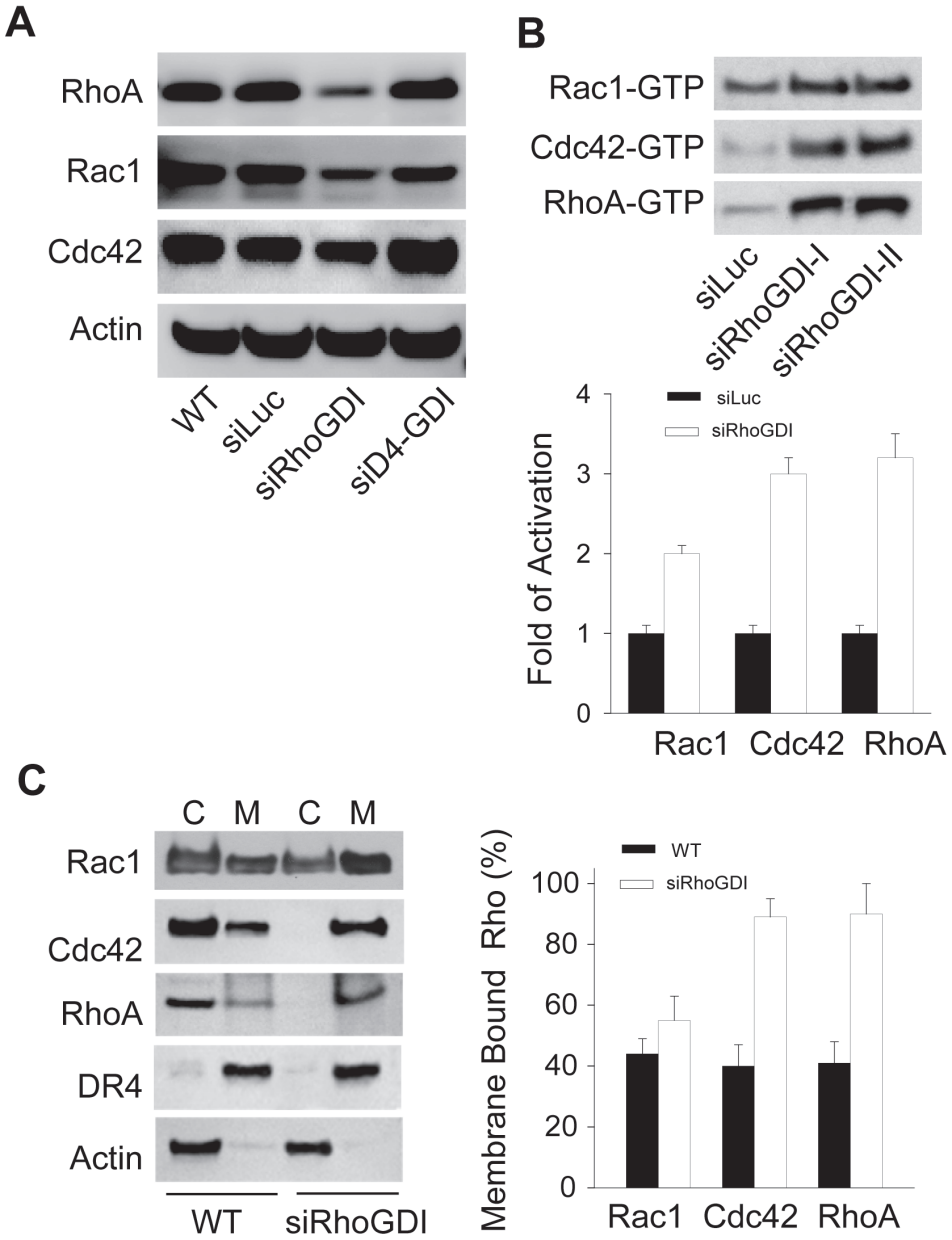
Loss of RhoGDI leads to constitutive activation of Rho GTPases **A.** Immunoblot analysis of total cellular RhoA, Rac1, and Cdc42 protein levels in parental MB-231 and derivative cell lines. **B.** Immunoblot analysis of GTP-bound Rho GTPases. Activation of endogenous Rac1, Cdc42, and RhoA were detected by effector pull-down assays using protein-binding domains (PBD) as lures (see Methods). Quantification of siLuc (black bars) and siRhoGDI (white bars) blots was accomplished by densitometry (mean ± SD of three independent experiments). *p < 0.01 compared to siLuc MB-231 cells. **C.** Subcellular localization of endogenous Rho GTPases. Cells were lysed and fractionated into crude membrane and cytosolic fractions before immunoblotting for each indicated Rho GTPase. Effectiveness of subcellular fractionation was confirmed by immunoblotting for cell surface death receptor-4 (DR4) and cytosolic actin. *Right panel*, quantification of WT (black bars) and siRhoGDI (white bars) blots was accomplished by densitometry (mean ± SD of three independent experiments). *p < 0.01 compared to WT cells.

To understand how RhoGDI knockdown stimulated tumor growth (Fig. 1B), we first determined the status of Rho GTPases in RhoGDI-deficient cells. RhoGDI is known to form stable complexes with individual Rho GTPases such as Rac1, Cdc42, and RhoA; thereby stabilizing the Rho proteins and keeping them in an inactive state within the cytosol. When RhoGDI is under expressed, Rho GTPases are released and undergo proteolytic degradation [15]. Consistently, we observed a slight decrease in total Rho GTPase protein levels in RhoGDI depleted cells when compared to parental cells (Fig. 2A). Despite the lowered total protein expression, the levels of active Rho GTPases (GTP-bound) were significantly higher in RhoGDI-deficient cells compared to control cells (Fig. 2B). Similar results were obtained using two distinct siRhoGDI RNA sequences (siRhoGDI-I and siRhoGDI-II), indicating specificity of silencing RhoGDI transcript. The increase in Rho GTPase activity coincided with a translocation of Rho GTPase from the cytosol to membrane compartments as indicated by sub-cellular fractionation and immunoblotting (Fig. 2C). Compared to parental cells, RhoGDI-depleted cells showed a significant decrease in cytosolic Rac1, Cdc42, and RhoA along with an increase in membrane localization. Among the three Rho GTPases tested, both RhoA and Cdc42 were almost exclusively found in the membrane fraction of siRhoGDI cells. Consistent with a modest increase in active GTP-bound Rac1 (Fig. 2B), Rac1 membrane translocation was also less pronounced compared to Cdc42 and RhoA (Fig. 2C). This is in agreement with our previous data that shows Rac1 but not Cdc42 or RhoA binds to RhoGDI and D4-GDI with comparable affinities [4]. Because D4-GDI expression was not affected in siRhoGDI cells, our results indicate D4-GDI can likely provide a compensation for the loss of RhoGDI in regulating Rac1 subcellular localization and activity. Together this demonstrates that loss of RhoGDI triggers a constitutive activation of multiple Rho GTPases and their translocation from the cytosol to membrane compartments.

### COX-2 protein is upregulated in RhoGDI-deficient cells

**Figure 3:**
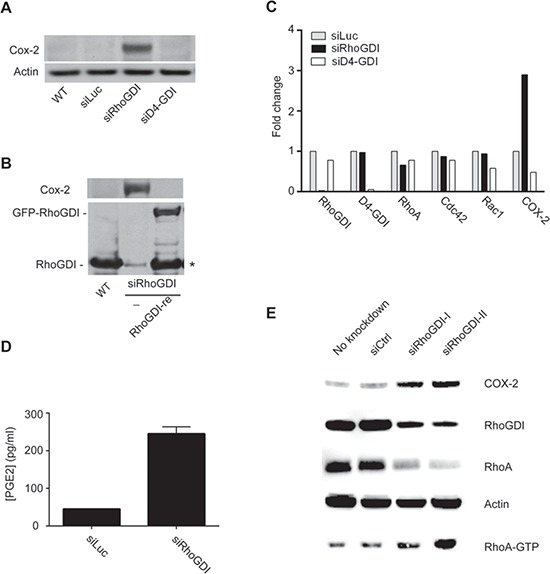
RhoGDI deficiency induces an upregulation of COX-2 protein expression **A.** Immunoblot analysis of COX-2 protein expression in parental MB-231 cells and stable clones expressing siLuc, siRhoGDI, or siD4-GDI. **B.** COX-2 and RhoGDI immunoblots depicting restoration of RhoGDI expression suppresses COX-2 protein to basal levels. Stable siRhoGDI cells were transfected with a pEGFP-C3 plasmid encoding a functional RhoGDI mutant that is not susceptible to preexisting siRNA (see Methods), indicated by RhoGDI-re. * Proteolytic cleavage of the expressed GFP-RhoGDI fusion protein was detected. **C.** qPCR analysis for mRNA expression of RhoGDI, D4-GDI, RhoA, Cdc42, Rac1, and COX-2. Actin was used as a reference gene and fold changes were determined by normalizing to siLuc MB-231 cells. **D.** PGE_2_ ELISA plots for siLuc and siRhoGDI MB-231 cells. **E.** Immunoblot analysis of RhoGDI knockdown in HCA-7 colon carcinoma cells.

A body of evidence shows that Rho GTPases can regulate COX-2 protein expression [16–18] and that COX-2 is overexpressed in many types of cancer. Therefore, we evaluated COX-2 expression in siRhoGDI cells. Strikingly, western blot analysis revealed that siRhoGDI cells displayed significantly higher levels of COX-2 when compared to parental and siLuc control cells (Fig. 3A). The upregulation of COX-2 protein was selective to loss of RhoGDI as silencing D4-GDI did not increase COX-2 protein expression (Fig. 3A). To confirm a direct inverse correlation with RhoGDI and COX-2, we rescued RhoGDI expression in siRhoGDI cells by transfecting a plasmid encoding RhoGDI mutant cDNA. In this construct the siRhoGDI targeted region was mutated to prevent knockdown by the pre-existing siRhoGDI in the cells. When RhoGDI expression was restored, the level of COX-2 was reduced to basal levels as observed in parental cells (Fig. 3B). qPCR analysis revealed that RhoGDI knockdown also resulted in a strong increase in COX-2 mRNA expression (Fig. 3C). Because COX-2 is responsible for the biosynthetic production of prostaglandin, a key molecule in tumorigenesis [11], we compared prostaglandin production for siLuc and siRhoGDI MB-231 cells. In agreement with increased COX-2 protein and mRNA expression, RhoGDI knockdown also led to increased prostaglandin production judged by PGE_2_ ELISA (Fig. 3D). Similar observations were made for HCA-7 colon carcinoma cells where targeted knockdown of RhoGDI increased COX-2 protein expression (Fig. 3E). These data suggest that RhoGDI may be directly involved in the regulation of COX-2 expression at least in some cancer cells.

**Figure 4:**
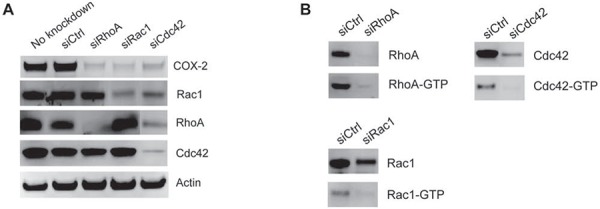
Increased COX-2 expression is triggered by Rho GTPase activation **A.** COX-2, RhoA, Rac1, and Cdc42 immunoblot analysis of siRhoGDI MB-231 stable cells transfected with siRNA targeting RhoA, Rac1, and Cdc42. **B.** Immunoblot analysis of active GTP-bound Rho GTPase after siRNA knockdown.

We were interested in determining if the elevated COX-2 expression observed in siRhoGDI MB-231 cells was a result of the constitutive Rho GTPase activation. To this end, RhoA, Rac1, and Cdc42 expression was knocked down by transient siRNA transfection in the stable siRhoGDI MB-231 cell line. Notably knockdown of individual Rho GTPase led to a significant reduction in COX-2 in RhoGDI-depleted cells (Figure 4A). Importantly we confirmed that siRNA knockdown led to a decrease in total and active GTP bound Rho GTPase (Fig. 4B). This confirms that enhanced Rho GTPase activity accomplished by RhoGDI depletion leads to increased levels of COX-2, which in turn affects prostaglandin levels that can induce cancer cell proliferation.

### RhoGDI protein expression is downregulated as a function of breast cancer progression

**Figure 5:**
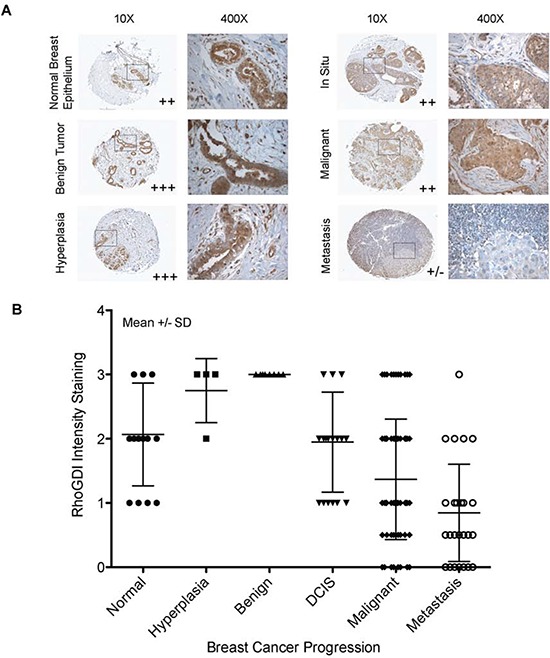
RhoGDI protein expression as a function of human breast cancer progression **A.** Immunohistochemical staining of RhoGDI in breast cancer progression tissue array (BR2082, US Biomax). Representative images are from tissues of normal breast epithelium, benign tumor, hyperplasia, in situ, and metastasis tumors (lymph node positive). **B.** RhoGDI intensity staining was quantified as a function of breast cancer progression. Each tissue sample was analyzed by two certified pathologists and was given a semi-quantitative score based on staining intensity (y-axis), 0- no staining, 0.5- very weak staining, 1- weak staining, 2- medium staining, and 3- strong staining. All samples were grouped according to disease stage (x-axis). The average combined staining intensity scores were then plotted (mean ± SD).

To test the physiological relevance of the above observations, we screened for RhoGDI protein expression in primary breast tumors using tumor tissue microarrays consisting of a panel of 165 tissue samples with different cancer disease stages. A monoclonal antibody specific to RhoGDI protein was used for immunohistochemistry analysis. As a negative control, a duplicate array was stained with normal rabbit serum. Fig. 5A illustrates representative immunostaining images for different samples during the progression of breast cancer. In normal breast epithelium and benign tumors RhoGDI was localized to areas around the ducts, where strong staining was observed. By contrast, in samples taken from patients with carcinoma in situ, malignant tumors, or metastatic disease, there was a pattern of weak and diffuse RhoGDI staining (Fig. 5A). After analyzing all 165 tissue samples, we found a strong downward trend for RhoGDI expression in breast cancer samples from invasive malignant and metastatic tumor isolates as compared to either carcinoma in-situ or normal healthy tissue (Fig. 5B).

### Inhibition of Rho GTPase and COX-2 leads to decreased breast cancer cell viability

**Figure 6:**
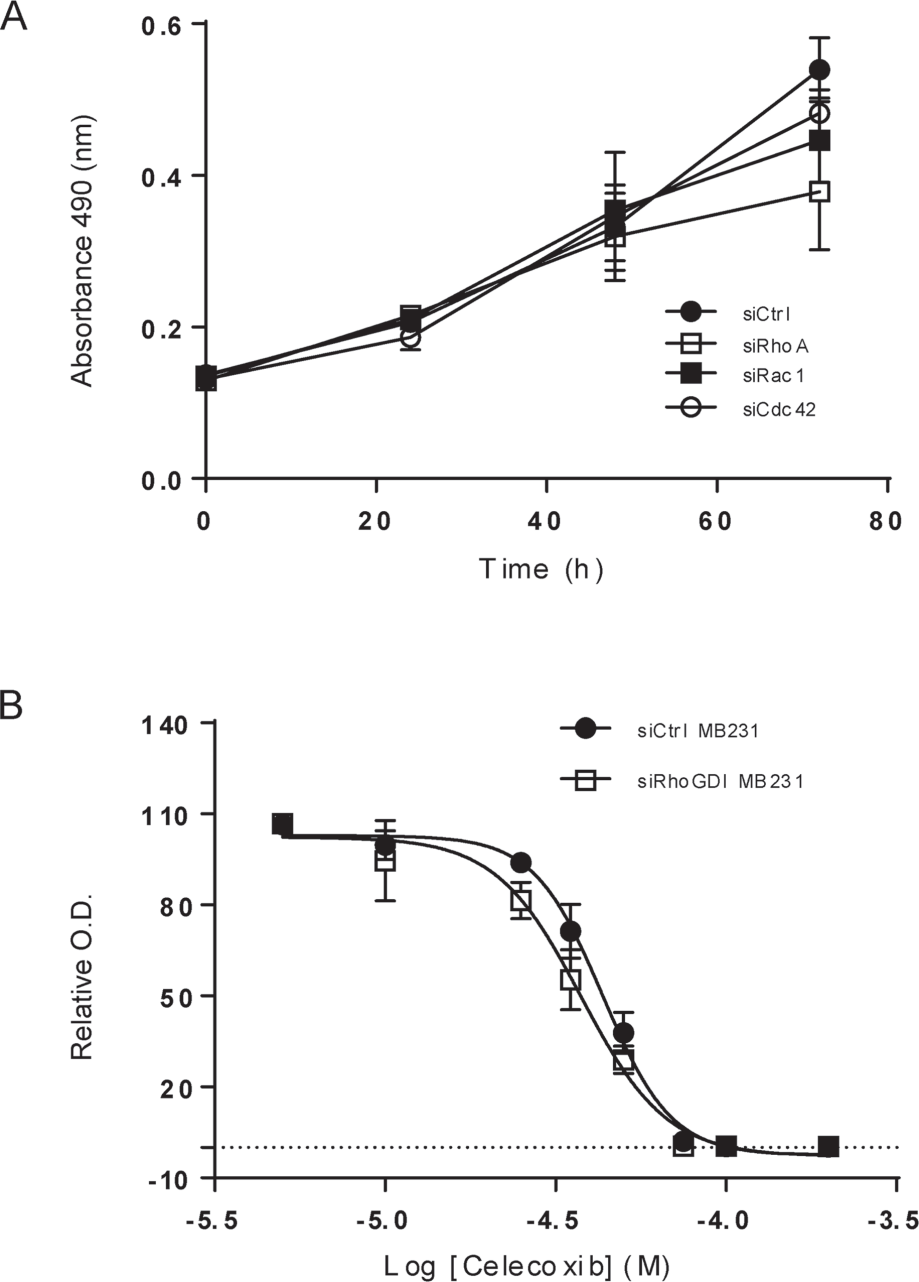
Blockade of Rho GTPases and COX-2 decreases cell viability in breast cancer cells **A.** siRhoGDI MB-231 cells were grown onto 96-well plates and were transiently transfected with control siRNA or siRNA specific to RhoA, Rac1, and Cdc42, respectively. Cell viability was determined using MTS reagent. **B.** Cells were treated with Celecoxib at the indicated concentrations at 37°C for 48 h. EC_50_ values were estimated to be 43 μM for siLuc MB-231 cells and 38 μM for siRhoGDI MB-231 cells.

To test the therapeutic potential of inhibiting Rho GTPases and COX-2, we determined cell viability upon blockade of each individual molecule. As shown in Fig. 6A, specific knockdown of RhoA, Rac1, or Cdc42 had a similar effect in suppressing cell proliferation in siRhoGDI MB-231 cells. Additionally, pharmacological inhibition of COX-2 led to a dose dependent decrease in cell viability for siLuc- and siRhoGDI MB-231 cells (Fig. 6B).

## DISCUSSION

Accumulating evidence shows that RhoGDI and D4-GDI are aberrantly expressed in certain types of human cancers [19, 20]. For example while ovarian cancers have been shown to display high levels of both RhoGDI [21, 22] and D4-GDI [23] compared to normal tissue; RhoGDI has been shown to be under expressed in both primary non-small cell lung cancer (NSCLC) and malignant gliomas [24, 25]. Similarly, D4-GDI expression is reported to be significantly lower in primary bladder carcinoma when compared to normal tissue [26–28] and has also been identified as a suppressor of metastasis. Interestingly in pancreatic cancer, studies have shown that elevated expression levels of D4-GDI led to increased cellular invasion [29]; while in other work, impaired RhoGDI function led to an increase in pancreatic cancer cell proliferation and metastatic potential [30]. Conflictingly in hepatocellular carcinoma (HCC), RhoGDI was found to promote cell proliferation and migration [31]; while in other reports RhoGDI expression was decreased in half of the HCC cases investigated [32]. Further characterization of 147 HCC samples revealed that loss of RhoGDI was correlated to a worsening of clinical prognosis [33].

Conflicting data has also been reported for RhoGDI expression in breast cancer cells. Initially, Fritz et al. reported an increased RhoGDI protein expression in breast cancer tumor tissue when compared to normal tissue from four separate patients [6]. In contrast, Jiang et al. observed a decrease in RhoGDI expression in tumors by quantitative RT-PCR and immunohistochemistry staining, when comparing 120 breast tumor samples with 32 normal tissue samples [5]. In agreement with Jiang et al., we showed that loss of RhoGDI had a pronounced effect in promoting tumor growth *in vivo* (Fig. 1B). This effect is in sharp contrast to our previous data showing that loss of the homologous D4-GDI led to abrogation of tumor growth *in vivo* [4]. Together this suggests that RhoGDI (RhoGDI-1) and D4-GDI (RhoGDI-2) can have opposing roles in the regulation of breast cancer progression. One explanation may be related to the differences in RhoGDI and D4-GDI in binding selectivity for Rho GTPases such as RhoA, Cdc42, and Rac1. Although all Rho GTPases tested were shown to have elevated activation and an increased membrane translocation in response to genetic knockdown of RhoGDI, we observed that RhoA and Cdc42 are significantly more sensitive to the loss of RhoGDI when compared to Rac1. These differences are likely due to that fact that Rac1 is known to also bind to D4-GDI, which appears to provide a compensation for the loss of cellular RhoGDI protein. We have previously reported that D4-GDI preferentially binds to Rac1, whereas RhoGDI binds Rac1, RhoA, and Cdc42 with comparable affinity [4]. Together, our results indicate that reduced expression of RhoGDI can generate a distinct Rho GTPase activation pattern which can influence specific downstream effectors such as COX-2.

COX-2 is an inducible enzyme, activated by IL1β, IL6, or TNFα [34], that is responsible for converting arachidonic acid to prostaglandin and other eicosanoids [35, 36]. Prostaglandins are lipid mediators that trigger biological functions by activating the G-protein coupled receptors [37]. Prostaglandins have been shown to affect cell proliferation, cell death, and tumor invasion in colon, breast, and lung cancer [11]. Additionally, COX-2 has been extensively shown to be overexpressed in many pancreatic, breast, colorectal, and lung cancers and is often associated with poor prognosis [7–11]. Several studies have shown a role of Rho GTPases in regulating COX-2 expression. For example, constitutive expression of active RhoB in HCA-7 and LS-174 colon carcinoma cells led to increased levels of COX-2. Similarly, over expression of RhoA, Rac1, and Cdc42 all led to induced COX-2 expression in NIH3T3 cells, MDCK epithelial cells, and HT29 colon cancer cells through a NF-κB dependent mechanism [18]. Subsequently, it was determined that oncogenic activation of Ras, Rac1, and RhoA coordinately upregulate COX-2 protein levels in NIH 3T3 cells [16]. In general agreement, our data demonstrate that loss of RhoGDI leads to activation of multiple Rho GTPases, such as RhoA and Cdc42, and Rac1 to a lesser extent, which is directly associated with an increase in COX-2 gene and protein expression.

COX-2 activity is tightly regulated in order to control the cellular levels of prostaglandin. During tumorigenesis, aberrant synthesis of prostaglandin occurs [38, 39]. Although rigorously tested, we were unable to consistently stimulate cell proliferation of MB-231 cells *in vitro* solely by the addition of arachidonic acid or prostaglandin. We in fact observed that at high micromolar concentrations these lipids are toxic to the cells. Although we believe that the elevated levels of COX-2 and the corresponding increased prostaglandin production are key determinants in the augmented tumor growth rates of siRhoGDI MB-231 xenografts, we believe the tumor microenvironment itself is also an essential factor. It is well documented that prostaglandins can induce tumor progression through exploitation of multiple mechanisms [40]. Importantly prostaglandins can function as both autocrine and paracrine bioactive lipids which can trigger the release of growth factors, pro-inflammatory mediators, and angiogenic factors from tumor cells and also from surrounding epithelial or stromal cells. These events aid in switching the local microenvironment from normal to tumor supporting, leading to additional recruitment of immune and tumor infiltrating cells. This further stimulates the production of eicosanoids and growth factors which contribute to tumor growth and evasion of immune system attack. The complexity of the tumor microenvironment is possibly the reason that differences in cell proliferation rates were not observed for siRhoGDI and parental cells when cultured under *in vitro* conditions despite their significant differences in xenograft tumor models (Fig. 1). A recent report showed that silencing of RhoGDI expression in breast cancer cell lines altered the expression of several other proteins [41], suggesting that RhoGDI may exert its function through regulation of multiple pathways. Studies are underway in our laboratory to identify molecular targets downstream of RhoGDI using genomic and proteomic approaches.

**Figure 7:**
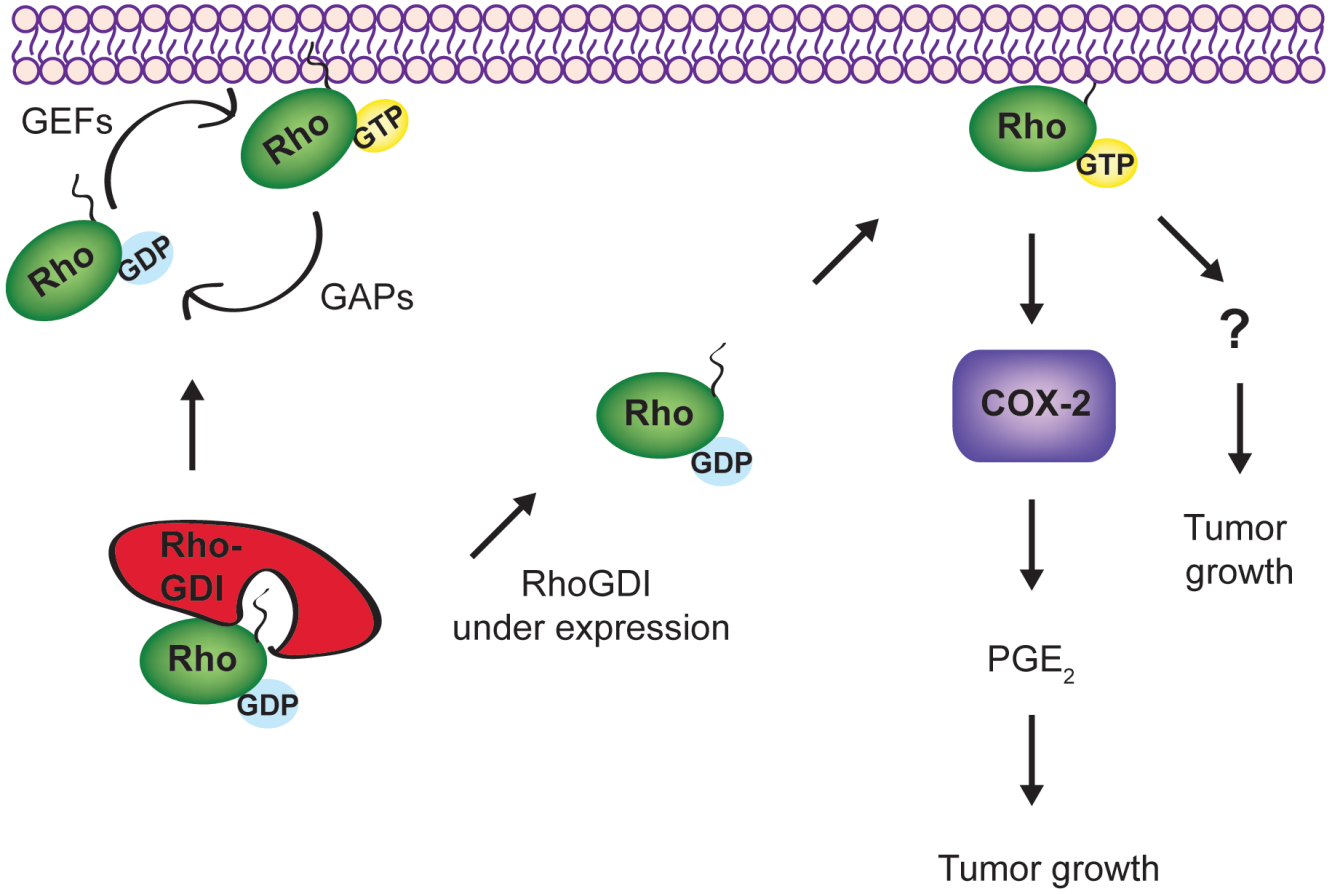
A possible signaling network links RhoGDI, Rho GTPases, and COX-2 In cells, RhoGDI forms stable complexes with each Rho GTPase at 1:1 stoichiometry; thereby stabilizing Rho GTPases and keeping them in an inactive form (GDP-bound) within the cytosol. In forming stable complexes, RhoGDI also inhibits the interactions between Rho GTPases and other regulatory proteins, such as guanine nucleotide exchange factors (GEFs), GTPase activating proteins (GAPs), and downstream effector proteins. When RhoGDI is under expressed, which is found to occur in malignant and metastatic breast tumors (Fig. 4), Rho GTPases (e.g., RhoA and Cdc42) appear to become constitutively active and translocate to membrane compartments. The activated Rho GTPases upregulate COX-2 expression and other signaling events yet to be characterized, contributing to the accelerated tumor growth. Blockade of Rho GTPases or COX-2 activity appears to inhibit cell growth or induce cell death in treated cells.

Although the detailed mechanisms remain to be determined, RhoGDI deficiency promotes breast cancer growth at least partly through activation of Rho GTPases and subsequent upregulation of COX-2 signaling (see illustration in Fig. 7). We have supported our findings through immunohistochemical analysis of RhoGDI protein expression in 165 breast tissue samples, representative of different cancer disease stages. Our study revealed that although strong RhoGDI expression was observed in benign tumors when compared to normal breast tissue, a marked decrease in RhoGDI expression was observed in both malignant tumors and metastatic lesions. A similar bi-phasic expression pattern has also been observed for D4-GDI during breast cancer progression [42, 43].

In summary, RhoGDI appears to play an important role in breast cancer through modulation of Rho GTPase-dependent cellular pathways. The downregulation of RhoGDI protein during breast cancer progression warrants additional studies to evaluate its potential use as a prognosis biomarker. RhoGDI deficiency appears to result in constitutive activation of multiple Rho proteins, which subsequently upregulates numerous pathways (e.g., COX-2) promoting uncontrolled cell growth. This work supports the potential significance of developing therapeutic inhibitors specific to Rho GTPases and COX-2 for the treatment of breast cancer. Relatedly, it has recently been determined that while chemotherapy can induce apoptosis it can also trigger the production of prostaglandin E_2_ (PGE_2_) which can cause a repopulation of cancer stem cells (CSCs) [44]. This finding highlights the importance of COX-2 signaling in chemotherapy resistance. Indeed it was determined that pharmacological inhibition of COX-2 by Celecoxib led to decreased chemoresistance in preclinical studies [44]. Currently, multiple clinical trials are underway to investigate the potential of celecoxib as a combinational therapy for the treatment of breast cancer [45–47]. Our data suggests that RhoGDI downregulation could be a critical mechanism of breast cancer progression. Loss of RhoGDI appears to trigger constitutive activation of multiple Rho GTPases (e.g., Cdc42 and RhoA) and subsequent upregulation of COX-2 activity. These data warrant further studies to explore the therapeutic potential of inhibiting Rho GTPases and COX-2 in the treatment of breast cancers.

## MATERIALS AND METHODS

### Cell lines and reagents

The MDA-MB-231 human breast cancer cell line was obtained from the American Type Culture Collection (Manassas, VA). Its derivatives stably expressing siRNA against human RhoGDI (siRhoGDI), D4-GDI (siD4-GDI), or firefly luciferase (siLuc) transcripts were generated by transfection with pRNA-U6.1 plasmids and have been previously described [12, 48]. The siRhoGDI sequence corresponds to ^401^GGAAAGGCGTCAAGATTGA^419^ of human RhoGDI gene transcript. For rescue studies, stable siRhoGDI clones were transfected with a pEGFP-C3/RhoGDI plasmid encoding a mutant RhoGDI cDNA sequence in which RhoGDI gene was mutated (^403^AAAGGCGTCAAGATTGAC^420^ to ^403^AAGGGAGTAAAAATCGAT^420^) [12]. Such a mutation does not change the amino acid sequence of the encoded RhoGDI protein, but it prevents destruction of exogenous mRNA by the preexisting siRNA in the siRhoGDI cells. Cells were grown in DMEM/F-12 (1:1) medium (Mediatech, Herndon, VA) containing 5% fetal bovine serum (FBS), 4 mM glutamine, 50 μM β–mercaptoethanol, and 1 mM sodium pyruvate at 37°C and 5% CO_2_ in air. The stable cell lines were maintained in complete medium supplemented with hygromycin at 450 μg/mL. All cell lines were periodically (~ 3 months) tested for the absence of mycoplasma contamination. HCA-7 cells were obtained from AddexBio (San Diego, CA) and were maintained in DMEM media containing 10% FBS.

Antibodies specific to human D4-GDI, RhoGDI, Rac1, Cdc42, and RhoA were obtained from BD Pharmingen (Lexington, KY). Anti-COX-2 antibodies were from Cell Signaling (Beverly, MA). Prostaglandin E_2_ ELISA kits were from Cayman Chemical (Ann Arbor, MI).

The siRNA duplexes targeting RhoA, Rac1, and Cdc42 were purchased from Life Technologies (Carlsbad, CA). The siRNA duplexes targeting RhoGDI were purchased from Life Technologies (Carlsbad, CA) and Sigma (St. Louis, MO). Transient transfection was carried out using lipofectamine RNAiMAX from Life Technologies (Carlsbad, CA) according to the manufacturer's instructions.

### Cell proliferation assays

Cells were cultured in complete medium supplemented with 5% FBS on plastic dishes or flasks. At the indicated times, the numbers of live cells were determined by MTS assay from Promega (Madison, WI) according to the manufacturer's instructions.

### Matrigel culture

Matrigel culture was performed as previously described [48]. Briefly, cells (2 × 10^4^) were plated onto a thick layer (1 mm) of Matrigel in eight-well chamber slides (Nunc, Naperville, IL). Solidified Matrigel was covered with complete growth medium and incubated at 37°C and 10% CO2 in air. At the indicated times, cell morphology was analyzed by phase-contrast microscopy.

### Animal studies

Female athymic nude mice, purchased from national cancer institute (NCI), 4–6 weeks old, were used for all studies in accordance with a protocol approved by the FDA Institutional Animal Care and Use Committee (Protocol#WO-2006-50). Cells were grown to 70–80% confluency, harvested by trypsinization, and washed twice in PBS. For xenografting studies, 5 × 10^6^ cells were suspended in 100 μL of Hank's balanced salt solution (HBSS) and injected subcutaneously (s. c.) into the back of the mice (10 mice per group). Tumor growth was monitored at the indicated times by externally measuring tumor length (L) and width (W) with a caliper. Xenograft volume (V) was calculated by the following equation: V = (LxW^2^) × 0.5.

### Endogenous Rho GTPase activity

GTPase activity assay is based on the high-affinity binding of GTP-bound GTPases to their specific effector proteins [49]. GTP-RhoA was detected using GST-rhotekin-Rho binding domain (RBD). GST-PAK1 binding domain (PBD) was used to determine the levels of active Rac1 and Cdc42. Briefly, cells were grown to 80% confluence on plastic dishes in the presence of 5% FBS. Cells were then harvested and lysed in a buffer containing 50 mM HEPES, 150 mM NaCl (pH7.5), 1 mM EGTA, 1% Triton X-100, 10% glycerol, 10 mM MgCl_2_, and protease inhibitors. Equal amounts of cell lysates were incubated with agarose-immobilized GST-rhotekin or GST-PAK1 at 4°C for 30 min. The co-precipitates were subjected to immunoassays using antibodies specific to individual Rho GTPases.

### Western blot analysis

Cells (1 × 10^6^) were lysed in buffer containing 50 mM Tris-HCl (pH. 7.0), 2% SDS, and 10% glycerol and were incubated for 20 min at 95°C. Protein concentrations were estimated using the BCA protein assay from Pierce (Rockford, IL). Equal amounts of cell lysates (20 μg per lane) were resolved by electrophoresis using a 4–12% NuPAGE Bis-Tris gel (Invitrogen) and were transferred to nitrocellulose membranes (Millipore) for immunoblot analysis with an appropriate dilution of antibodies (1:1000 to 1:2000). When necessary, the membranes were stripped by Restore Western Blot Stripping Buffer from Pierce (Rockford, IL) and reprobed with appropriate antibodies. Immunocomplexes were visualized by chemiluminescence using ECL from Santa Cruz (Dallas, TX) or SuperSignal reagent from Pierce (Rockford, IL).

### qPCR analysis

qPCR assays were performed at a contract laboratory (Phalanx Biotech, Belmont, CA), using primers specific to RhoGDI, D4-GDI, RhoA, Rac1, Cdc42, and COX-2 respectively. Actin was used as a housekeeping gene. Briefly, 2 μg of total RNA from each cell line was used for reverse transcription (RT) using ABI High-Capacity cDNA reverse transcription kits. 20 μl RT products were diluted with 80 μl nuclease-free H_2_O to generate 5X-dilution RT products (20 ng/μl). Each reaction included 20 ng cDNA, 500 nM forward and reverse primers, and 1X Fast SYBR Green PCR Master Mix (Applied Biosystems, 4385612). Each sample was tested in triplicate. A BIO-RAD CFX Connect real-time PCR machine was used with the following program: 95°C for 20 sec and 39 cycles of 95°C for 5 sec followed by 60°C for 30 sec. BIO-RAD CFX Manager Version 3.0 software was used for experimental setup and data analysis. Target gene qPCR data was normalized to the reference gene. Fold change was calculated as follows, the delta Ct (cycle threshold) of the control gene was subtracted from the delta Ct of the target gene for each sample. Then according to the comparisons specified the resulting delta Ct values for each sample were subtracted from each other resulting in the delta delta Ct value. Then, fold change was calculated by taking 2^−ΔΔCt^.

### COX-2 activity assay

Evaluation of COX-2 mediated conversion of arachidonic acid into prostaglandin was performed using a prostaglandin immunoassay kit from Cayman Chemical (Ann Arbor, MI), according to the manufacturer's recommended protocol and as previously described [50].

### Tissue microarrays

Breast cancer tissue microarrays (TMAs; Cat No. BR2082) were obtained from US Biomax, Inc. (Rockville, MD). The TMA contains a total of 165 breast tissues specimens including 29 cases of metastatic carcinoma, 84 invasive carcinoma (malignant), 16 intraductal carcinoma, and 3 lobular carcinoma in situ (In Situ), 8 fibroadenoma (benign), 4 of hyperplasia, 21 adjacent normal tissue, and normal tissue, single core per case. All tissue samples were preserved in 10% phosphate buffered formalin (pH 7.4), embedded in paraffin, processed into sections. The tissue microarray sections were cut at 4 μm think. Individual cores are 1.5 mm in diameter, spaced 0.25 mm.

For immunohistochemistry analysis, tissue sections were deparaffinized in xylene, and rehydrated in gradients of alcohol and water. Endogenous peroxidase activity was blocked by incubating slides in 3% hydrogen peroxide at room temperature for 5 minutes. Antigen retrieval was performed in the antigen unmasking solution, H3300, for 30 minutes in a microwave oven. To reduce non-specific staining, slides were washed in phosphate buffered saline with Tween-20, followed by incubation in 2.5% normal horse blocking serum for 30 min. Blocked sections were incubated with Mouse monoclonal anti-RhoGDI antibody (BD Biosciences, Catalog No. 610255) (1:250 dilution) for 1 hour at room temperature. After washing three times, slides were incubated for 30 minutes with ImmPRESS reagent (Vector Laboratories) followed by incubation with the peroxidase substrate DAB solution (DAKO Cytomation) until desired stain intensity develops. Slides were counterstained with Hematoxylin and mounted with permanent mounting medium.

Qualitative/semi-quantitative scoring was independently done by two board certified pathologists. Staining intensities in cancer cells were compared with inflammatory cells (particularly lymphocytes) that are known to express high levels of D4-GDI. This was used as an internal positive control. The cells of stroma, collagen, and adipose tissues showed minimal or no staining and were used as an internal negative control. The staining intensities when compared to negative and positive controls on the same tissue core were defined as: negative or background level staining [0], weak staining [1], moderate intensity staining [2], and strong intensity staining [3]. The average staining intensity was calculated from the semi-quantitative scores given by each of the two pathologists or an average of duplicated cores accordingly.

### Statistical analysis

Statistical analyses were performed using ANOVA followed by Bonferroni's multiple comparison test or Student's *t* test.
